# Bio-nanocomplexes with autonomous O_2_ generation efficiently inhibit triple negative breast cancer through enhanced chemo-PDT

**DOI:** 10.1186/s12951-022-01706-0

**Published:** 2022-11-24

**Authors:** Zhihong Zeng, Zhou Wang, Simin Chen, Chang Xiao, Minzhuo Liu, Jie Zhang, Jialong Fan, Yanzhong Zhao, Bin Liu

**Affiliations:** 1 College of Biological and Chemical Engineering, Changsha University, Changsha, 410022 China; 2 College of Biology, Hunan University, Changsha, 410082 China; 3 Department of Health Management, The Third Xiangya Hospital, Central South University, Changsha, 410011 China

**Keywords:** Cerium oxide loaded nanoparticles (CeNPs), Triple-negative breast cancer, CS-1, PDT

## Abstract

As one kind of aggressive cancer, triple-negative breast cancer (TNBC) has become one of the major causes of women mortality worldwide. Recently, combinational chemo-PDT therapy based on nanomaterials has been adopted for the treatment of malignant tumor. However, the efficacy of PDT was partly compromised under tumor hypoxia environment due to the lack of sustainable O_2_ supply. In this study, CeO_2_-loaded nanoparticles (CeNPs) with peroxidase activity were synthesized to autonomously generate O_2_ by decomposing H_2_O_2_ within tumor region and reprogramming the hypoxia microenvironment as well. Meanwhile, the compound cinobufagin (CS-1) was loaded for inhibiting TNBC growth and metastasis. Moreover, the hybrid membrane camouflage was adopted to improve the biocompatibility and targeting ability of nanocomplexes. In vitro assay demonstrated that decomposition of H_2_O_2_ by CeO_2_ achieved sustainable O_2_ supply, which accordingly improved the efficacy of PDT. In turn, the generated O_2_ improved the cytotoxicity and anti-tumor migration effect of CS-1 by downregulating HIF-1α and MMP-9 levels. In vivo assay demonstrated that the combination of CS-1 and PDT significantly inhibited the growth and distance metastasis of tumor in MDA-MB-231 bearing mice. Thus, this chemo-PDT strategy achieved satisfactory therapeutic effects by smartly utilizing the enzyme activity of nanodrugs and special micro-environment of tumor.

**Supplementary Information:**

The online version contains supplementary material available at 10.1186/s12951-022-01706-0.

## Introduction

The International Agency for Research on Cancer estimates of cancer incidence and mortality show that breast cancer is the most commonly diagnosed cancer with the leading cause of death in females [1]. As a highly heterogenic and extremely fatal subtype, Triple-Negative Breast Cancer (TNBC) with poor prognosis and high relapse rate often leads to lung, liver, and brain metastasis [2, 3]. Moreover, the development of specific targeting drugs is difficult due to the lack of estrogen receptors, progesterone receptors, and absence of erbb2 (HER2) in TNBC [4]. Recently, different adjuvant platinum-based chemotherapy and BRCA mutation-directed therapy in metastatic tumors showed potential benefits yet incomplete pathology responses were often observed in patients [5–8]. Consequently, different regimens of therapy are required to address the high recurrence and distant metastasis of tumor.

Photodynamic therapy (PDT), a clinically approved tumor treatment method, can directly induce cellular damage to microvasculature and cell membranes by converting O_2_ from the ground state to reactive oxygen (ROS) [5–8]. As an emerging treatment modality, the minimal normal tissue toxicity, negligible systemic effects, long-term reduction in morbidity, and lack of intrinsic or acquired resistance as well as organ function-sparing effects have made it the potential to become an ideal TNBC treatment strategy [9]. However, as an O_2_-dependent method, PDT is incompatible under the hypoxic microenvironment of tumors in many cases [10, 11]. To overcome this problem, various O_2_-generating materials including MnO_2_ and perfluoro hexane were adopted to alleviate tumor hypoxia during the PDT treatment period by transporting limited O_2_ to the tumor tissue at a single time [12, 13]. However, as light exposure at the tumor site needs to be performed repeatedly during the whole course of PDT, designing oxygen vector with continuous O_2_ supply is necessary. CeO_2-_based nanoparticles (CeNPs) with peroxidase-like activity was reported to reversibly switch from Ce^4+^ to Ce^3+^ under weak acidic environment. Therefore, they can achieve O_2_ evolution in the presence of H_2_O_2_, which made it an ideal continuous O_2_ supplier [14]. In addition, Ce^3+^-dependent peroxidase-like activity mediated by the Fenton/Haber Weiss reaction over a wide range of pH and temperature was also found [15]. Moreover, CeNPs showed better stability compared with natural enzymes. These properties showed great potential for use in ROS-mediated tumor catalytic treatment by combining CeNPs-based PDT and other chemo drugs to further improve the sensitivity of therapy [16, 17].

Due to the complicated heterogeneity, enhancing PDT by improving the hypoxic microenvironment is not sufficient to inhibit the growth and migration of TNBC, which requires the assistance of other high-efficiency and targeting therapy strategies. Cinobufagin (CS-1), a bufanolide steroid secreted by the Asiatic toad *Bufo gargarizans*, is one of the important ingredients in the anti-cancer Chinese medicine of Chansu [18]. This compound evoked strong anti-tumor effects through IL-6/STAT3, PI3K/Akt, and MAPK pathways [19]. In addition, it can increase intracellular ROS and reduce reduced glutathione (GSH) levels by inhibiting glutathione reductase (GR) [20]. Therefore, considering the lack of estrogen receptors, progesterone receptors, and erbb2 (HER2) expression in TNBC [21], combining PDT with CS-1 using nanomaterial may be an ideal strategy for TNBC treatment. However, naked nanocomplexes can be rapidly cleared by the immune system after entering into blood circulation. Recently, cell membrane coating has become a strategy to improve nanoparticles bioactivity, including long circulation time and disease targeting. Among these membranes, the erythrocyte membrane significantly increased immune-escape ability [22], while tumor membrane can promote nanoparticle penetration into the core region of cancer through a homing effect [23]. Thus, the hybrid of cancer cell membrane and erythrocyte membrane endowed longer blood circulation and excellent isotype targeting ability of NPs for enhanced in vivo therapeutic efficacy.

**Fig. 1:**
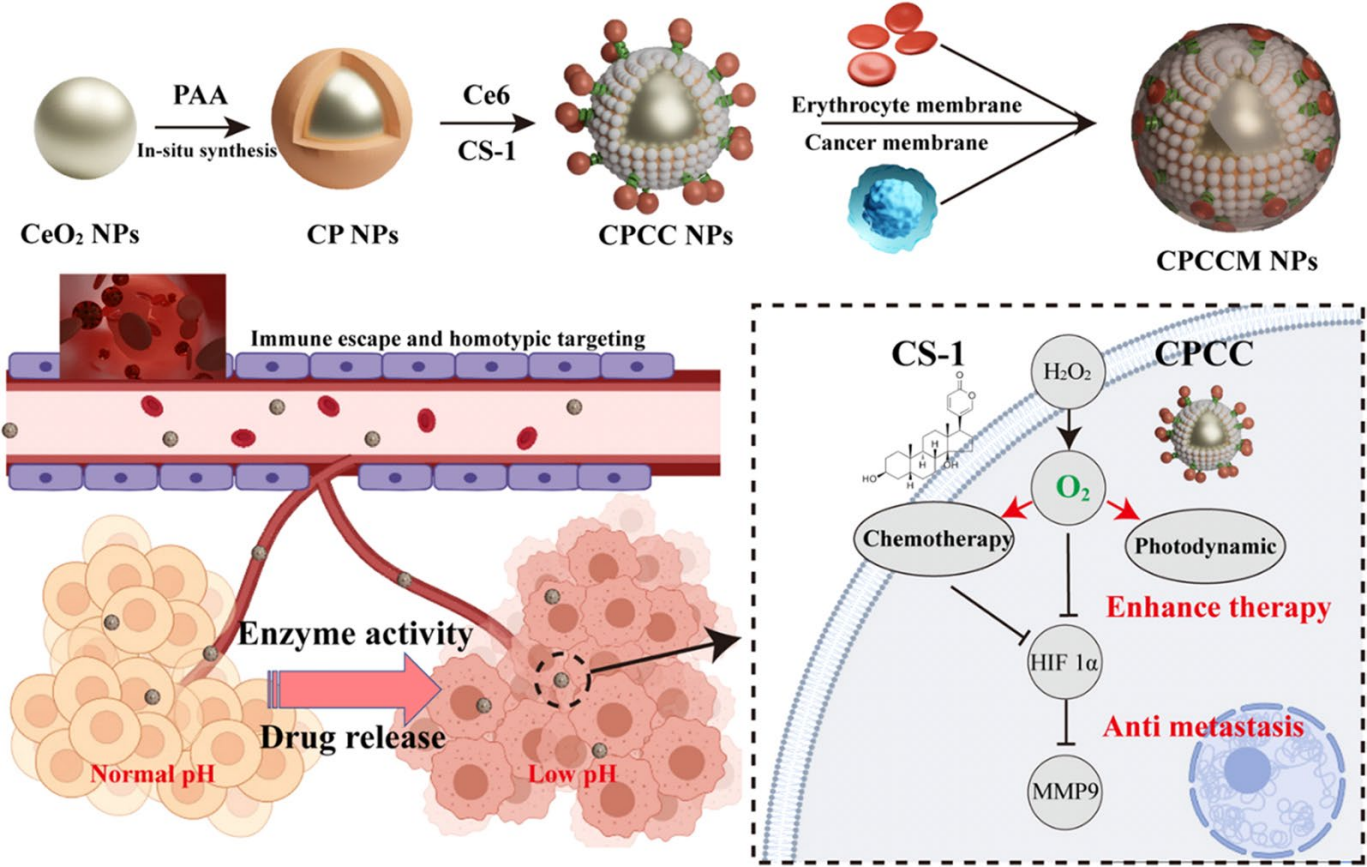
Schematic diagram of the designed strategy for the combined therapy of CPCCM NPs

In this work, a CS-1 loaded nanocomplexes (CPCCM) with hybrid membrane coating was developed to inhibit tumor growth and metastasis. The schematic diagram for the preparation of CPCCM NPs is shown as Fig. 1. The hybrid membrane was adopted to simultaneously endow immune-escape ability during the circulation and homologous targeting ability to the tumor region, as well. Once entering into tumor tissues, CPCCM NPs with peroxidase-mimic activity then decompose excess H_2_O_2_ into O_2_. The sustainable O_2_ supply can accommodate multiple PDT sessions over longer periods. Under NIR irradiation, O_2_ acts as a substrate for photosensitizers to produce highly toxic ^1^O_2_ to kill tumor cells. In theory, the CPCCM NPs with peroxidase activity and anti-metastasis ability can improve the efficacy against TNBC.

## Materials

Ce_2_(CO_3_)_3_ was gifted from Yiyang Hongyuan Rare Earth Co. Ltd; Polyacrylic acid (PAA, MW: 200 Da) was purchased from Shanghai Macklin Biochemical Co., Ltd. Chlorin e6 (Ce6) was purchased from Shanghai Yuanye Bio-Technology Co., Ltd (Shanghai, China). CS-1 was purchased from Herbest (Xi’an, China). Polysorbate-20, Dimethyl sulfoxide (DMSO), N-hydroxy-succinimide (NHS), 1-ethyl-3-(3-dimethylaminopropyl) carbodiimide (EDC) and 4′,6-diamidino-2-phenylindole (DAPI) were purchased from Sigma-Aldrich (MO, USA). ROS-ID Hypoxia/Oxidative Stress Detection Kit was purchased from Enzo Co., Ltd (New York, USA). Hoechst 33342, ROS kit, TUNEL detection kit and Calcein AM/Propidium Iodide (PI) kit were purchased from Yeasen biotech Co., Ltd (Shanghai, China). Cy5.5 fluorescence dye and Hematoxylin–Eosin staining kit were purchased from Beijing Solarbio Science & Technology Co., Ltd (Beijing, China). CCK-8 kit was purchased from TargetMol (MA, USA). Other chemicals and reagents were all analytical grades.

### Cell culture and animals

The MDA-MB-231 (human breast cancer cell line), 4T1 (mouse breast cancer cell line), SMC (human smooth muscle cell line), NIH-3T3 (embryonic mouse fibroblast line), RAW264.7 (mouse macrophages cell line) were purchased from Cell Bank of Xiangya Central Experiment Laboratory of Central South University. All cells were cultured in the Dulbecco’s modified Eagle’s medium (DMEM, HyClone, UT, USA) supplemented with 10% FBS (Gibco, PA, USA) and 1% Penicillin & streptomycin (PS, Invitrogen, CA, USA) at 37 °C under a 5% CO_2_ atmosphere. Female BALB/c and nude BALB/c mice were purchased from Hunan SJA Laboratory Animal Co., Ltd. All animal experiments complied with the guiding principles of “Declaration of Helsinki, DoH” were carried out according to the guidelines for the care and use of laboratory animals of Animal Care and Use Committee of Hunan University.

## Methods

### Preparation of polyacrylic acid modified cerium nanoparticles (CP)

5 mL solution containing 1 M Ce_2_(CO_3_)_3_ (with nitric acid treatment) were mixed with 10 mL polyacrylic acid (0.05 M). After ultrasonic treatment for 10 min, 30 mL of 30% ammonium solution were added and the mixture kept stirring for 24 h. The prepared solution was centrifuged at 4000 rpm for 30 min and washed with ultra-pure water to remove insoluble precipitation. The PAA-modified CeO_2_ NPs were obtained after centrifugation at 12,000 rpm for 30 min. After dispersing in the ultra-pure water and ultrasound treatment for 10 min, the solution was frozen to get powder.

### Preparation of chlorin e6 and CS-1 loaded cerium nanoparticles (CPCC)

For loading Ce6, 1 mL CeO_2_ NPs solution (1 mg/mL) were mixed with 0.6 mg of EDC and 1.2 mg of NHS. After stirring for 30 min, 1 mL of Ce6 solution (0.05 mg/mL) was added to the mixture and kept stirring for 24 h. Then, the mixture was centrifugated at 12,000 rpm for 10 min to remove the excess Ce6, EDC, and NHS. The finally obtained precipitation was resuspended in the deionized water.

For the CS-1 load, 1 mg of CS-1 was dissolved in 1 mL of dimethylsulfoxide (DMSO). Then, 0.1 mL CS-1 solution was added dropwisely into 1 mL (1 mg/mL) CeO_2_ NPs solution or Ce6 loaded CeO_2_ NPs. After stirring for 24 h, the solution was centrifugated at 12,000 rpm for 10 min to remove the excess CS-1. The obtained precipitation was resuspended in the ultra-pure water.

### Preparation of erythrocyte-cancer hybrid membrane (RBC-231)

RBC membranes were prepared following our previous report [23]. The whole blood collected from female BALB/c mice (6–8 weeks) was centrifuged at 3,500 rpm for 5 min at 4 °C. The precipitated RBCs were washed with cold 1× PBS for 3 times. Then, 0.25× PBS was added into cells and incubated on the ice for 30 min. The RBC membranes were obtained after centrifugation at 12,000 rpm for 5 min. After washing with cold 1× PBS for 3 times, the collected membranes were stored at – 80 °C.

MDA-MB-231 cell membranes were prepared following the instruction of the membrane protein extraction kit. In brief: Tumor cells washed with ice-cold PBS were suspended in the membrane protein extraction reagent A containing 1 mM PMSF for 15 min at 4 °C. After through 3 freeze–thaw cycles and 50 W sonication for 3 min, the mixture was centrifuged at 3000 rpm for 10 min at 4 °C. The supernatant was centrifuged at 13,000 rpm for 30 min at 4 °C to obtain cell membrane. After mixing the RBC membrane and MDA-MB-231 membrane with equal weight, the mixture was treated with ultrasonication for 10 min at 37 °C to complete membrane fusion.

### Preparation of the hybrid membrane coated CPCC (CPCCM)

The obtained CPCC was ultrasonic for 30 min to redisperse into monodisperse system. Then, the mixture of CPCC and hybrid membrane with a weight ratio of 2:1 was add into an ultrasonic bath for 5 min to obtain hybrid membrane-coated CPCC NPs(CPCCM NPs). The uncoated membrane was removed through centrifugation at 12,000 rpm for 30 min at 4 °C. The CPCCM NPs were washed with PBS until no protein was detected in the supernatants.

### Characterization of material and RBC-231 hybrid membrane

For characterization of material, the morphology was assessed using a high-resolution JEOL JEM-2100Plus TEM (Hitachi Scientific Instruments, Japan). The FT-IR spectra were recorded with a Thermo-Nicolet Nexus 6700 FT-IR spectrometer (USA). The UV–Vis spectra were measured with a DU800 spectrometer (Beckman Coulter Inc., USA). The size and zeta potential of nanomaterial were measured with a Zetasizer Nano analyzer (Malvern Nano Series, UK). The chemical states of the elements were measured with an X-ray photoelectron spectroscopy (XPS) (ULVAC-PHI Inc., Japan).

A fluorescence assay was illustrated to characterize the extent of membrane fusion. Briefly, the DiI labeled RBCM and DiO labeled 231 M were mixed with equal weight. The fluorescence of the hybrid membrane was imaged under confocal laser scanning microscope (CLSM).

SDS-PAGE was employed to characterize the integrity of membrane proteins. Briefly, RBCM, 231 M, HM, and CPCCM were lysed with membrane protein extraction reagent B containing 1 mM PMSF. The protein concentration of all samples was detected using the BCA Kit, followed by proteins (20 μg) denature at 95 °C for 10 min. The proteins were separated in the 12% SDS-PAGE gel and stained using Coomassie Brilliant Blue solution.

### Detection of peroxidase-like activity and PDT of CPCCM NPs in vitro

1 mM TMB and 5 mM H_2_O_2_ were sequentially added into 10 μg/mL of CPCCM NPs solution. The absorbance values of samples at 652 nm (the characteristic peak of ox-TMB) were monitored at different time points. H_2_O_2_ of series concentration was used for kinetic study of peroxidase activity of CPCCM NPs.

The dissolved O_2_ concentration was detected using dissolved O_2_ meter. Briefly, 500 μg CPCCM NPs were added to 10 mL 1 mM H_2_O_2_ solution (pH6.8) and the dissolved O_2_ concentrations were measured at different time point. In parallel sample, the amount of H_2_O_2_ consumption was determined after adding ammonium molybdate (final concentration of 2.4 mM) by recording the absorbance values at 330 nm.

The generation of ^1^O_2_ by Ce6, CPCC, and CPCCM was detected using the trapping agent of TEMP. Briefly, 1 mL of Ce6, CPCCM, and CPCC NPs (with equivalent concentration of 5 μg/mL Ce6) was mixed with 100 μL of TEMP (640 μM). After with 660 nm laser irradiation (0.2 W/cm^2^, the ^1^O_2_ signal was immediately detected by the ESR. In addition, the quantitative detection of ^1^O_2_ was detected using SOSG probe. Ce6, CPCC, and CPCCM NPs (equivalent concentration of 5 μg/mL Ce6) were mixed with SOSG (5 μM) dissolved in PBS (pH 7.4) following with 660 nm laser irradiation (0.2 W/cm^2^). The fluorescence intensities of samples at 530 nm were recorded at predetermined time intervals (Ex = 488 nm).

### Loading and releasing behavior in vitro

To determine loading capacity (LC) and encapsulation efficiency (EE) of CS-1 and Ce6, different amounts of CS-1 and Ce6 were redispersed in CP NPs solution. The loading method is consistent with the above CPCC NPs synthesis protocol. Final nanomaterials were centrifuged at 12,000 rpm for 10 min to collect the supernatant. The absorbance values of 296 nm for CS-1 and 410 nm for Ce6 were measured for constructing standard concentration curve. The LC and EE were calculated using the following formulas: LC (%) = (Mt-Mu)/Mp × 100%, and EE (%) = (Mt-Mu)/Mt × 100%.

Where MT represents the total mass of CS-1 and Ce6 for loading, MU represents the unencapsulated mass and MP represents the mass of CP NPs.

To monitor CS-1 release behavior, 2 mg CPCC NPs with 1.56 mg CS-1 were dispersed in PBS solution (1 mL) (pH7.4/6.8). The supernatant containing released CS-1 was collected at different time points by centrifuging at 8000 rpm for 10 min, simultaneously, the content of CS-1 was detected by UV–Vis spectrophotometer. For the group treated with NIR radiation, the suspended materials were radiated (660 nm, 0.2 W/cm^2^, 5 min) with 3 lasers on/off cycles.

### Biosafety and biocompatibility assay

The hemolysis assay was performed using the whole blood of healthy female BALB/c mice. Blood samples were centrifuged at 3000 rpm at 4 °C for 5 min and washed 3 times with PBS. 50 μL of 4% erythrocytes (v/v) was dissolved in 950 μL PBS (pH 7.4) with CP, CPCC, and CPCCM NPs (concentrations varied from 25 to 300 μg/mL) and incubated at 37 °C for 4 h. All samples were centrifuged at 3000 rpm at 4 °C for 5 min. The absorbance values (540 nm) of supernatants were measured on the UV–Vis spectrophotometer. Hemolysis rates were calculated using Eq. (1). In parralle, the separated erythrocytes were used for morphological imaging under microscope.

1 $${\text{Hemolysis}}\,{\text{(\% ) = }}\left( {{{\text{I}} \mathord{\left/ {\vphantom {{\text{I}} {{\text{I}}_{0} }}} \right. \kern-\nulldelimiterspace} {{\text{I}}_{0} }}} \right) \times 100\%$$

where I means the absorbance of supernatant containing erythrocyte suspension and NPs and I_0_ means the absorbance of the whole erythrocytes-lysed water.

Platelet aggregation assay: Platelet-rich plasma prepared from the whole blood of BALB/c mice was mixed with CP, CPCC, CPCCM NPs (50 μg/mL), or thrombin (5 μg/mL) solution. After incubation for 1 h at 37 °C, the absorbance values of 650 nm were measured. Meanwhile, the samples containing plasma and thrombin or PBS (9:1, v/v) were used as positive and negative controls, respectively.

Cytotoxicity assay. NIH-3T3 and MDA-MB-231 cells were cultured in the 96-well plate (5 × 10^4^ cells/ well) for 24 h. Then, CP, CPCC, and CPCCM NPs with different concentrations (25 to 200 μg/mL) were incubated with cells for 24 h before the addition of 100 μl MTT (0.5 mg/mL). After 4 h, MTT was replaced by DMSO. The absorbance values of cells at 490 nm were measured for cell viability assay according to Eq. (2).

2 $${\text{Cell Viability (\% ) = }}\left( {{{{\text{OD}}_{{490\,{\text{nm/sample}}}} } \mathord{\left/ {\vphantom {{{\text{OD}}_{{490\,{\text{nm/sample}}}} } {{\text{OD}}_{{490\,{\text{nm/control}}}} }}} \right. \kern-\nulldelimiterspace} {{\text{OD}}_{{490\,{\text{nm/control}}}} }}} \right) \times 100\%$$

### Functional test of erythrocyte-cancer hybrid membrane

CLSM was used to evaluate the targeting ability of CPCC@Lip, CPCC@231, and CPCC@RBC-231. Membrane-coated CPCCM NPs (Ce6: Ex/Em = 400/653 nm) were used to estimate the cellular uptake efficacy in vitro. SMC and MDA-MB-231 cells were cultured in the 6-well plates with cover slip (1 × 10^6^ cells/well), respectively. After 24 h, PBS washed cells were separately incubated with CPCC@Lip, CPCC@231, and CPCC@RBC-231 NPs (50 μg/mL) for 6 h, respectively. Then, cells were washed with PBS before CLSM imaging. The immune evasion assay was performed with the above method by using RAW264.7 cells as target cells.

### Anti-tumor performance in vitro

Cell uptake assay: MDA-MB-231 cells were cultured into 12-well plates with cover-slip (5 × 10^4^ cells/well) for 24 h. The medium was then replaced with fresh medium containing CPCCM NPs (equivalent to 5 μg/mL Ce6) and incubated for 2, 4, 6, and 8 h. The nuclei were stained with Hoechst 33342 and images of cells were captured under CLSM.

Cell viability assay: MDA-MB-231 cells seeded in the 96-well plate (5 × 10^3^ cells/well) and cultured for 24 h. The hypoxic environment was constructed according to the instructions of the Anaero Pack-Anaero kit. Then, different materials were incubated with cells for 24 h before the addition of 100 μL of MTT (0.5 mg/mL). After 4 h, excess MTT was replaced by the DMSO and the absorbance values of samples were measured at 490 nm.

Live/dead staining: The hypoxic environment was constructed according to the instructions of the Anaero Pack-Anaero kit. The MDA-MB-231 cells were cultured into 12-well plates with cover-slip (5 × 10^4^ cells/well) for 24 h before the medium containing PBS, CPM, CS-1, Ce6, CPCM, and CPCCM NPs (CP, 50 μg/mL; Ce6, 2.5 μg/mL; CS-1, 5 μg/mL) was substituted with fresh medium. The cells were irradiated with 660 nm laser (0.2 W/cm^2^) for 3 min. After treating for 48 h, the MDA-MB-231 cells were stained with Calcein AM/Propidium Iodide for 5 min for imaging under CLSM.

ROS assay: MDA-MB-231 cells in 12-well plates with round cover slip (5 × 10^4^ cells/well) were cultured for 24 h. Then, the medium was replaced by the fresh medium containing PBS, CPM, CS-1, Ce6, CPCM, or CPCCM (CP, 50 μg/mL; Ce6, 2.5 μg/mL; CS-1, 5 μg/mL). After 6 h, the cells were irradiated with 660 nm laser (0.2 W/cm^2^) for 3 min, followed by the addition of DCFH-DA. The hypoxic environment was constructed according to the instructions of the Anaero Pack-Anaero kit. MDA-MB-231 cells washed with PBS were imaged under CLSM 30 min later.

For ^1^O_2_ generation and PDT enhancement assay: MDA-MB-231 cells were seeded into 12-well plates with cover slip (5 × 10^4^ cells/well) and incubated with PBS, CPM, CS-1, Ce6, CPCM or CPCCM (CP, 50 μg/mL; Ce6, 2.5 μg/mL; CS-1, 5 μg/mL) for 6 h. Then, cells were exposed to 660 nm laser irradiation for 3 min (0.2 W/cm^2^). Cyto-ID^®^ Hypoxia/Oxidative Stress Detection regent was added to the plates and incubated for 0.5 h for confocal imaging. The fluorescence signal intensities of the hypoxia probe (Ex/Em = 590/670 nm), and ROS probe (Ex/Em = 490/525 nm) and Hoechst 33,342 (Ex/Em = 346/460 nm) in the cells was measured using spectrofluorometer.

### Tumor mouse model

For the orthotopic breast cancer model, 100 μL PBS/Matrigel mixture containing 1 × 10^7^ MDA-MB-231 cells was injected into the fourth inguinal mammary fat pad on the left of healthy female BALB/c nude mice (4–6 weeks old). Tumor size was measured with caliper and tumor volume was calculated by the standard formula 0.5 × L × W^2^, where L is the long diameter and W is the short diameter.

### Body imaging and biodistribution of CPCCM NPs

200 μL free Ce6, CPCC, and CPCCM NPs (2.5 mg/kg Ce6 equivalent) solutions were injected intravenously into the female BALB/c mice (n = 3 per group). Blood samples were obtained from the retro-orbital plexus of mice at different time intervals (0.1, 1, 2, 4, 6, 8, 12, and 24 h). The blood sample was centrifuged at 3500 rpm for 10 min at 4 °C to obtain the plasma. 100 μL plasma from each sample was imaged using IVIS kinetics optical system (PerkinElmer, CA) to illustrate the fluorescence signal of Ce6.

For distribution of CPCCM in vivo: 14 days after orthotopic tumor implantation, mice were intravenously injected with equal volumes of free Ce6 or Ce6-labeled CPCC and CPCCM NPs (5 mg/kg of Ce6) (n = 3 per group). The whole bodies of mice were imaged at different time points using IVIS kinetics optical system. The major organs (heart, liver, spleen, lung, and kidney) and tumors were collected 48 h post-administration for imaging immediately under the same system.

### Anti-tumor performance in vivo

The healthy female BALB/c nude mice (4–6 weeks old) were subcutaneously injected with MDA-MB-231/Luc tumor cells, as described above. When the tumor volumes reached 50 mm^3^, mice were randomly divided into PBS group, CS-1 group, Ce6 + L group, CPCM + L group, and CPCCM + L treatment group (n = 5 per group). Mice were injected intravenously with the same doses of Ce6 (2.5 mg/kg) or CS-1 (1 mg/kg). 4 h later, the mice were locally irradiated with 660 nm laser (0.2 W/cm^2^) for 5 min. The body weights and tumor volumes of mice were monitored and recorded on the day of 0, 2, 4, 6, 8, 10, 12, 14, 16, 18, and 20. The primary tumor of each mouse was detected by bioluminescence imaging on the 20th day. The collected tumors and main organs were fixed in 4% paraformaldehyde for H&E and TUNEL staining. Meanwhile, blood samples of each group were collected for biochemical and hematological assay.

### Antitumor invasion and metastasis in vivo

4T1/Luc tumor model was established by subcutaneously injecting tumor cells into the female BALB/c mice (normal immune system, 4–6 weeks old). When the tumor volumes reached 50 mm^3^, they were randomly divided into PBS group, CS-1 group, Ce6 + L group, CPCM + L group, and CPCCM + L treatment group. They were intravenously injected with the same doses of Ce6 (2.5 mg/kg) or CS-1 (1 mg/kg) and with or without local laser irradiation (660 nm, 0.2 W/cm^2^, 5 min) 4 h after injection. After 20 days, the drug administration was stopped and the change in tumor volumes was observed. Tumors and main organs were collected for H&E and immuno-histochemical (IHC) staining using HIF-1α and MMP-9 antibodies. The total number of metastatic nodules in the lung and liver was counted to evaluate the anti-metastatic effects of CPCCM (n = 3 per group).

### Statistical analysis

Data were expressed as mean ± standard deviation (SD). T-test or One-way (ANOVA) test by using the GraphPad Prism 8.0.2 software was applied to test the significance between groups, where significant differences were defined as *p < 0.05, **p < 0.01, ***p < 0.001.

## Results and discussion

### Preparation and characterizations of CPCCM NPs

**Fig. 2:**
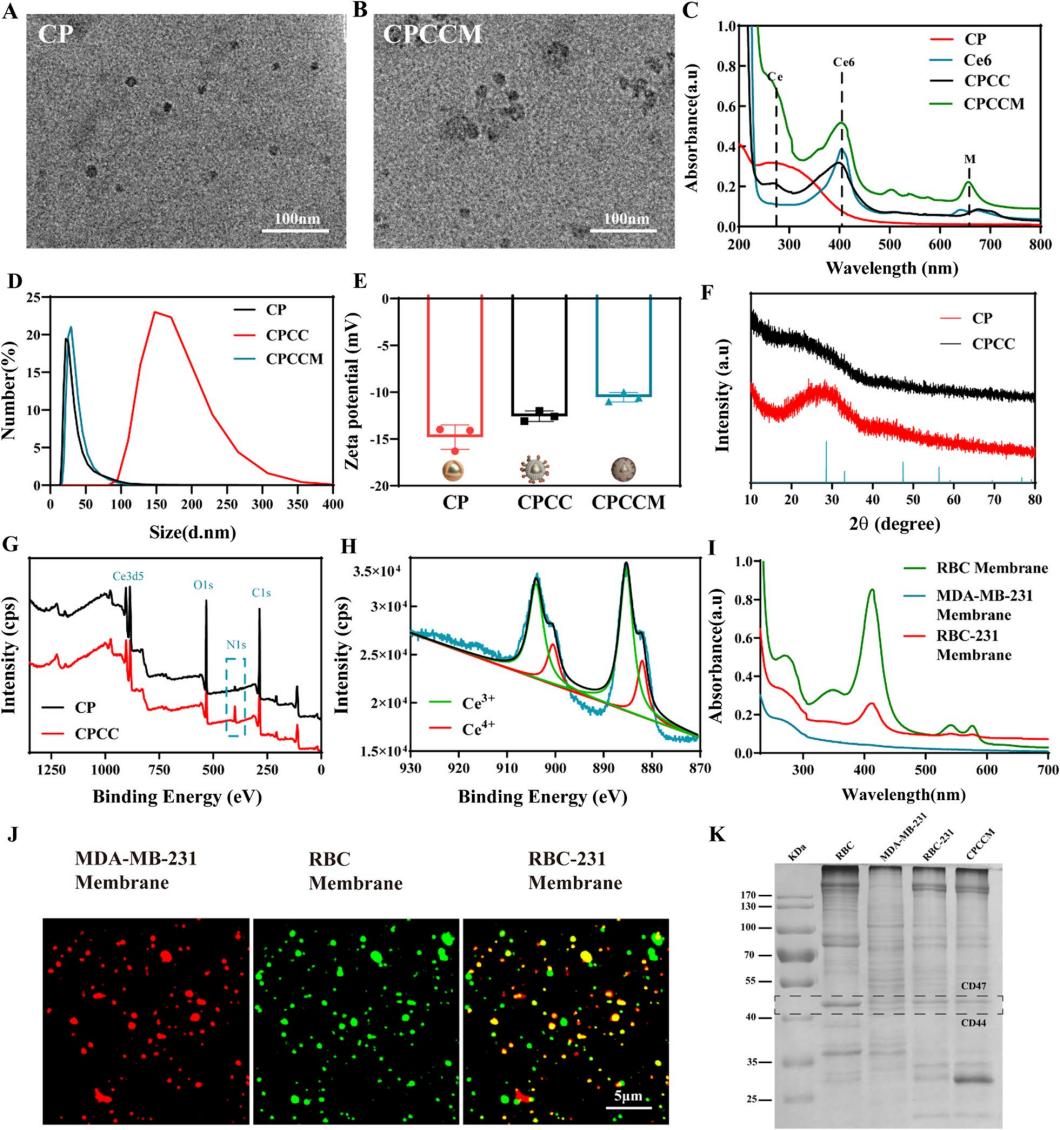
Preparation and Characterizations of CPCCM NPs. **A**, **B** TEM image of CP NPs and CPCCM NPs. **C** UV–Vis absorbance spectra of CP, Ce6, CPCC, and CPCCM. **D** The particle size distribution of CP, CPCC, and CPCCM. **E** Zeta potential analysis of CP, CPCC, and CPCCM, Mean ± SD, n = 3. **F** XRD spectrum of CP and CPCC NPs. **G** XPS spectrum of CP and CPCC NPs. **H** XPS analysis to show the chemical valence of Ce^3+^ and Ce^4+^ on the surface of CPCC NPs. **I** UV–Vis spectra of RBC, MDA-MB-231, and RBC-231 membrane samples. **J** Confocal fluorescent microscopy images of different membrane samples (red: RBC membrane, green: MDA-MB-231 membrane). **K** Protein characterization of RBC and MDA-MB-231 membrane samples by SDS-PAGE analysis. Data were shown as mean ± SD, n = 3

Using the facile PAA in-situ coating method [25], the CP NPs (CeO_2_@PAA) with an average size of 15 ± 5 nm were synthesized (Fig. 2A). Then, CS-1 and Ce6 were encapsulated on the surface of PAA by chemical modification, respectively. After coating with hybrid membrane (M), the size of CPCCM NPs (CeO_2_@PAA@CS-1/Ce6@M) became 30 ± 5 nm (Fig. 2B). UV–Vis spectra indicated characteristic absorption peaks of CS-1 at 275 nm and bio-membrane at 400 nm, respectively, apart from a strong absorption peak at 250–300 nm of Ce, (Fig. 2C). The average size of the prepared CPCC NPs (CeO2@PAA@CS-1/Ce6) increased to ~ 140 nm (Fig. 2D), due to the agglomeration caused by the hydrophobic CS-1 and outer layer Ce6 of CPCC NPs. The particle size of CPCCM showed that the CPCC agglomerates could be re-dispersed by ultrasonic coating of hydrophilic hybrid membrane. During the synthesis of CPCCM NPs, the surface charge decreased from − 11.2 mV (CP NPs) to − 14.9 mV due to the negative charge of CS-1 and the bio-membrane (Fig. 2E). XRD showed Main peaks of CP and CPCC (Fig. 2F). XPS spectra further indicated the typical peak of CeO_2_ NPs at the binding energy of 885.08 eV corresponding to Ce3 d5, while a typical peak (at the binding energy of 286.4 eV) ascribed to C1s was observed due to the PAA modification. Similarly, enhanced typical peak ascribed to N1s of CPCC NPs was found after loading CS-1 and Ce6 (Fig. 2G). In addition, the XPS spectra showed the multiplicity of final states in the Ce 3d photoionization process, which can dissolve into two multiples of Ce^4+^ and Ce^3+^. Obviously, oxidative states of Ce^4+^ (46.7%) were observed in CPCC NPs (Fig. 2H). This unique composition with Ce^4+^ endowed the CPCC NPs with peroxidase activity [24].

To illustrate the process of membranes fusion, UV -Vis spectra indicated characteristic absorption peaks of RBC membranes at 420 nm, which also observed in RBC-231 membranes (Fig. 2I). In addition, we performed another experiment using Dil and DiO dyes to separately label two kinds of cell membranes. The two kinds of dye-labeled membranes were mixed with equal weight. After with ultrasonication treatment for 30 min, the samples were observed under fluorescence scope. Figure 2J indicated bright yellow fluorescent signal, the overlap of red and green fluorescence, in the fused sample, due to the efficient fusion between them. SDS-PAGE analysis indicated that CPCCM NPs maintained membrane proteins of the two cell types, which is similar to those of hybrid membrane Importantly, CD44 and CD47, both the key membrane proteins in RBC and MDA-MB-231 cells were revealed (Fig. 2K). This result suggested the successful preparation of CPCCM NPs.

### Peroxidase activity in vitro

**Fig. 3:**
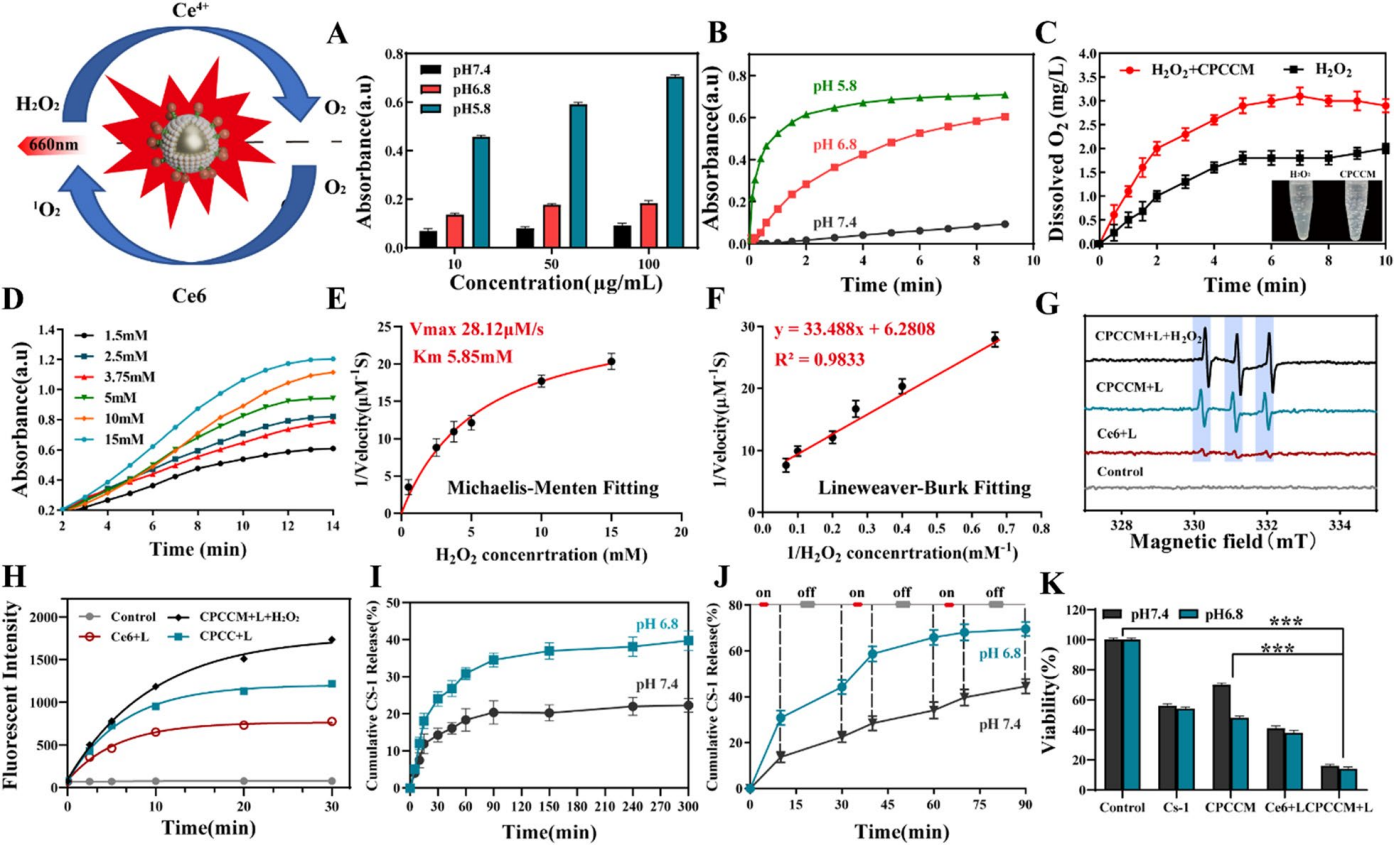
Peroxidase activity of CPCCM NPs. **A**, **B** UV–vis spectra of TMB (1 mM) with CPCCM at different pH values [H_2_O_2_] = 1 mM. **C** O_2_ concentration measurement by Dissolved Oxygen Meter in PBS (pH = 6.8) with CPCCM (50 μg/mL) and H_2_O_2_ (1 mM). **D** Absorbance of 50 μg/mL CPCCM NPs with the addition of 1.5–15 mM H_2_O_2_. **E** Michaelis–Menten kinetics of CPCCM NPs. **F** Lineweaver–Burk plotting of CPCCM NPs. **G** ESR analysis of ^1^O_2_ capture by TEMP (640 μΜ). **H** Singlet oxygen (^1^O_2_) generation ability is determined by SOSG (5 μΜ). **I** The CS-1 release from CPCCM NPs at pH 7.4 and pH 6.8. **J** The CS-1 release from CPCCM NPs under 660 nm laser irradiation (0.2 W/cm^2^). **K** Cell viability of MDA-MB-231 cells treated with different pH and CPCCM NPs (50 μg/mL) under 660 nm laser irradiation (0.2 W/cm^2^). Data were shown as mean ± SD, n = 3. ANOVA was used to assess statistical significance. ***p < 0.001

It was reported that Ce^4+^ can quickly oxidize TMB into blue oxTMB with a maximum absorbance peak at 652 nm, we then detected whether CPCCM NPs still possess peroxidase -activity. By changing the pH value and CPCCM concentration, we found that the absorbance value of, oxTMB increased in pH and CPCCM concentration-dependent manners (Fig. 3A). Moreover, weak acid environment greatly accelerated the reactive speed catalyzed by CPCCM. The absorbance value of sample reached the plateau after reacting for 3 min at pH 5.8. Compared with that of pH 7.4, the time was shortened about 60–80% (Fig. 3B). This pH-dependent activity is extremely beneficial for realizing the reaction in the tumor environment.

By mimicking the tumor acid environment (pH 6.8–6.4), CPCCM NPs were first placed in the solution (pH 6.8) to test the efficiency of O_2_ generation. Figure 3C indicated that O_2_ concentration reached 3.08 mg/L in 1 mM H_2_O_2_ solution in 10 min, the result of which reflects the efficient transformation of H_2_O_2_ into O_2_. By performing kinetic study of CPCCM catalyzed reaction (Fig. 3D), we found that the catalytic reaction between CPCCM NPs and H_2_O_2_ obeys the typical Michaelis–Menten kinetics. The Michaelis constant (Km) and maximal reaction velocity (Vmax) were calculated to be 5.85 mM and 28.12 μM/s, respectively (Fig. 3E, F). This result demonstrated that CPCCM NPs can reach half of the maximal catalytic activity even under 5.85 mM H_2_O_2_, which is urgent for efficient tumor therapy. To illuminate the ^1^O_2_ generation efficiency of CPCCM under laser irradiation, ESR assay, a direct method for radicals’ detection, was used to identify active free radicals by using 2,2,6,6-tetramethylpiperide (TEMP) as trapping probe of ^1^O_2_ (1:1:1, triplet signal). Figure 3G showed that simple Ce6 in anaerobic water with O_2_ pre-depletion showed weak triplet signal after laser irradiation. However, the excitation of ^1^O_2_ was enhanced when the Ce6 was loaded on the surface of CPCC NPs. The ^1^O_2_ production rate of CPCCM NPs contained solution reached the highest in the presence of H_2_O_2_. In addition, the fluorescent probe Singlet Oxygen Sensor Green (SOSG) assay showed the fluorescence signal caused by Ce6, CPCC, and CPCCM NPs with the assistance of laser increased in a time-dependent manner (Fig. 3H).

The modification of PAA is conducive to the loading and controlled release of drugs. According to the ratio of CS-1 and CP feeding ratio of 1:1, the encapsulation rate of the drug reached 76.53% and it will gradually increase with the increase of CP (Additional file 1: Fig. S1). In addition, by investigating the effect of pH on the CS-1 release, we found that the release rate of CS-1 from CPCCM after 5 h is 39.78% (pH 6.8), which is significantly higher than that of normal physiological environment (release rate of 22.31% at pH7.4) (Fig. 3I). Laser irradiation accelerated the drug release, which was reflected by the release rate of 72.84% within 90 min (Fig. 3J). After co-incubating MDA-MB-231 with CPCCM under different conditions, it was found that CPCCM showed stronger cell-killing effect at pH 6.8 (Fig. 3K).

### Hemocompatibility assay of CPCCM NPs

**Fig. 4:**
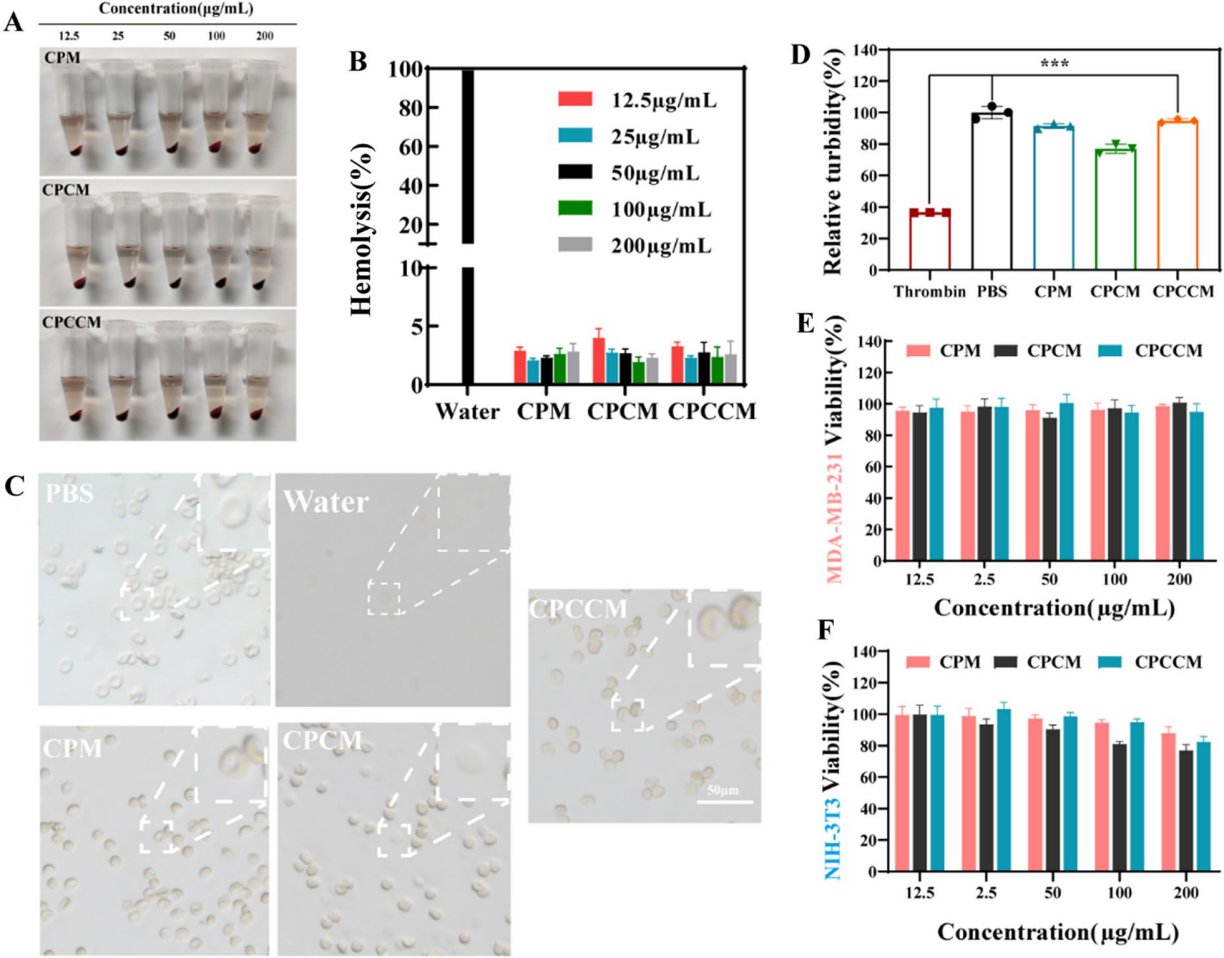
Biocompatibility evaluation. **A** The hemolysis images of red blood cells with CPM, CPCM, and CPCCM (12.5–200 μg/mL) treatment. **B** Hemolysis rates of NPs by UV–vis measured at 540 nm. **C** The microscopy image of red blood cells treated with CPM, CPCM, and CPCCM (50 μg/mL). **D** Platelet aggregation assay of CPM, CPCM, and CPCCM (50 μg/mL). **E** Cell viability assay of MDA-MA-231 cells and (F) NIH-3T3 cells treated with CPM, CPCM, and CPCCM (12.5–200 μg/mL). Data were shown as mean ± SD, n = 3. ANOVA was used to assess statistical significance, ***p < 0.001

The NPs can cause serious toxicity through interaction with erythrocyte cells, platelets, and immunoglobulins in the blood. To evaluate the practical application of CPCCM NPs in vivo, several methods were adopted to investigate their hemocompatibility in vitro. As shown in Fig. 4A, B, the negligible effect of 200 μg/mL CPCCM NPs on the hemolysis of erythrocyte cells, which was reflected by the low hemolysis rate (less than 5%). Meanwhile, Fig. 4C showed the normal biconcave disc form of erythrocyte cells treated with CPCCM NPs, which was significantly different from the aqueous solution treatment (cell rupture). We then investigated the effect of CPCCM NPs on platelet coagulation another essential indicator of hemocompatibility. Figure 4D indicated a small amount of coagulation of platelets treated with CPCC NPs. However, the coagulation rate reduced to ~ 5% for CPCCM NPs. This result demonstrated that bio-membrane coating can improve the hemocompatibility of NPs. Finally, MTT assay was adopted to study the cytotoxicity of nanomaterials (CP, CP@Ce6, and CPM@Ce6 NPs) without CS-1. After incubating cells with CP, CP@Ce6, and CPM@Ce6 NPs for 24 h, the cytotoxicity was assessed. Figure 4E and F indicate that the viability of MDA-MB-231 cells is higher than 90% even with 200 μg/mL CPM@Ce6 treatment. Although the viability of NIH-3T3 cells decreased in a dose-dependent manner, the viability was still maintained at 98.7% at the recommend dose (50 μg/mL). This result showed the high safety profile of CPM@Ce6 NPs for normal cells without laser irradiation.

### Function of erythrocyte-cancer hybrid membrane

**Fig. 5:**
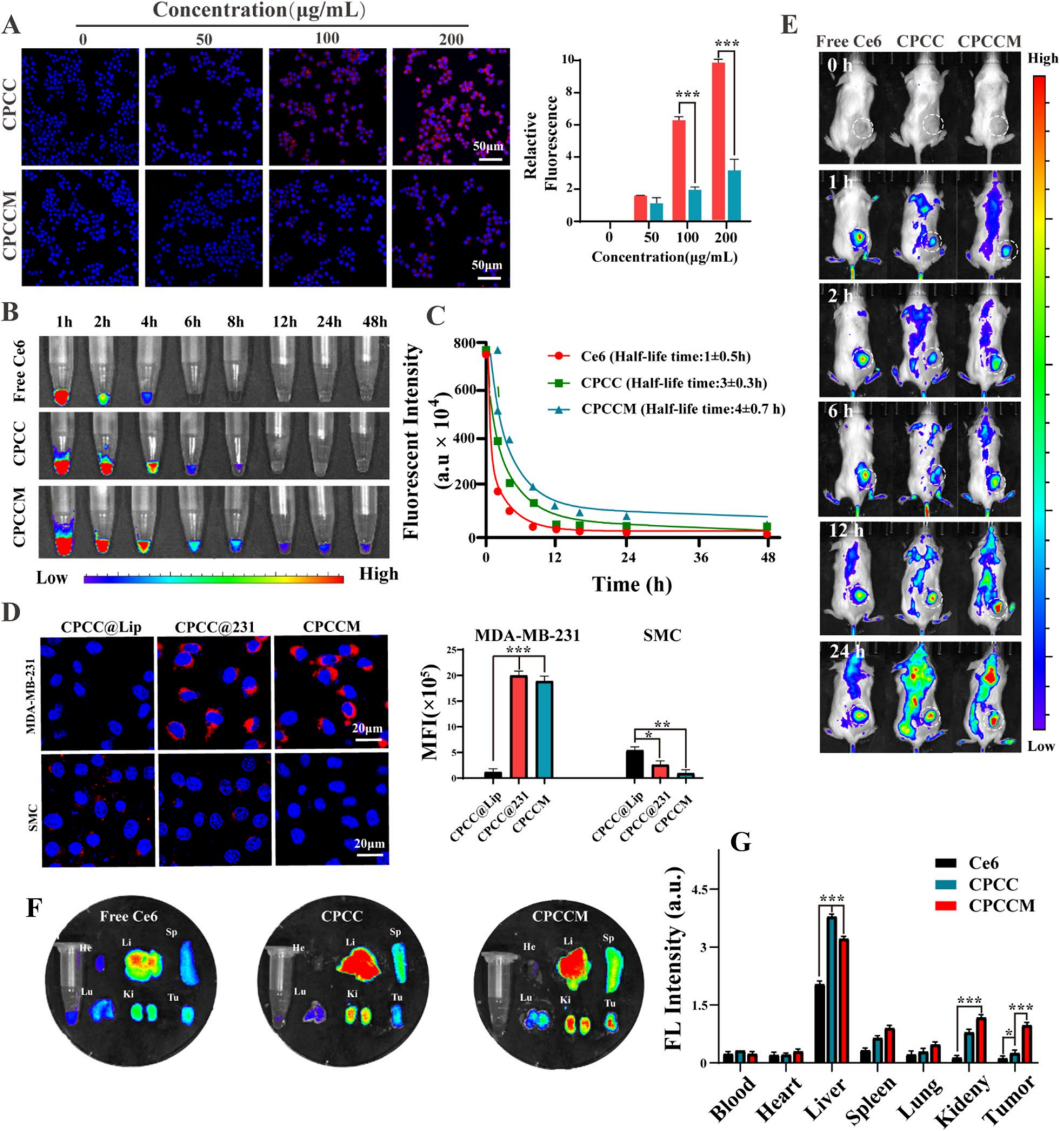
Function of erythrocyte-cancer hybrid membrane. **A** Confocal fluorescence imaging and quantitative analysis of CPCC and CPCCM NPs (50 μg/mL) in RAW264.7 cells (Bule: nucleus, red: Ce6). **B** Blood fluorescence intensity (Ce6: Ex/Em = 400/653 nm) of Ce6, CPCC, and CPCCM (2.5 mg/kg) over time. **C** Pharmacokinetic curves of Ce6, CPCC, and CPCCM. **D** Confocal fluorescence imaging and quantitative analysis of CPCC NPs (50 μg/mL) coated with the different membrane in MDA-MB-231 and SMC cells. **E** Bio-distribution of CPCC and CPCCM NPs (2.5 mg/kg) at 0, 1, 2, 6, 12, 24 h. **F** Fluorescence distribution of major organs after 48 h post-injection (2.5 mg/kg CPCC and CPCCM NPs). **G** Fluorescence intensity quantitative analysis of major organs after 48 h post-injection. Data were shown as mean ± SD, n = 3. ANOVA was used to assess statistical significance. *p < 0.05, ***p < 0.001

The immune-escape ability of CPCCM NPs was investigated using murine RAW264.7 macrophages as models. Figure 5A showed a significant reduction of the red fluorescence signal in cells with CPCCM NPs treatment, compared to bare CPCC NPs. Quantitative analysis showed that the macrophages phagocytosis rate reduced 70.3%. This result indicated that CPCCM NPs can evade the recognition by macrophages due to the expression of “don’t eat me” protein receptors (e.g., CD47) of erythrocyte membranes, which is helpful for extending blood circulation time. We further calculated the blood retention time of NPs by observing the fluorescence intensity of mouse blood in vivo (Fig. 5B). As expected, free Ce6 was eliminated rapidly from blood circulation in 1 h. Bare CPCC NPs with large size of ~ 140 nm was retained in blood for additional 2 h, which was attributed to the enhanced permeability and retention effect (EPR effect). However, the circulation half-time of CPCCM NPs was prolonged to 4.7 h, which was 1.5-fold that of bare CPCC NPs (Fig. 5C). The above results suggested that hybrid membrane could promote immune evasion to prolong the blood circulation of CPCCM NPs. Next, the uptake by tumors cells (MDA-MB-231 and SMC cells) of different membrane-coated NPs (CPCC@Lip, CPCC@231 NPs, CPCCM NPs) was investigated. We found that CPCC@231 and CPCCM NPs have significantly increased fluorescence intensity in MDA-MB-231 cells compared to CPCC@Lip NPs, indicating the enhanced targeting capability of CPCCM NPs. In contrast, weak fluorescence signal was observed in non-homologous SMC cells treated with CPCC@231 and CPCCM NPs. Even, the red fluorescent of CPCCM-treated cells was negligible, which was attributed to the effect of erythrocyte membrane on the uptake inhibition of normal cells (Fig. 5D). The hybrid membrane coating on the NPs only enhanced the targeting ability of specific tumor cells but not for normal cells, the result which was beneficial for protecting normal cells. To verify the tumor retention ability of CPCCM NPs, different NPs were injected into tumor bearing mice to observe their biodistribution (Fig. 5E). The strong fluorescence signal of free Ce6 within the tumor was observed at 1 h and was rapidly cleared within 24 h. By contrast, the obvious fluorescence signal was observed for 12 h in the mice tumor following CPCC and CPCCM NPs administration. Then, the fluorescence intensity increased gradually and reached the plateau at 24 h. Major organs of mice were collected for imaging and the CPCCM NPs were found to greatly accumulate in the tumor tissue (Fig. 5F, G). Quantitative analysis showed that the hybrid membrane increased the cumulative amount in tumor by 23.7%, compared to CPCC NPs. In addition, CPCCM NPs enrichment in the liver was significantly reduced possibly due to the small size. Taken together, these results validated the extended retention and active-targeting effects of CPCCM NPs.

### O_2_-Enhancing PDT/chemotherapy of CPCCM + L

**Fig. 6:**
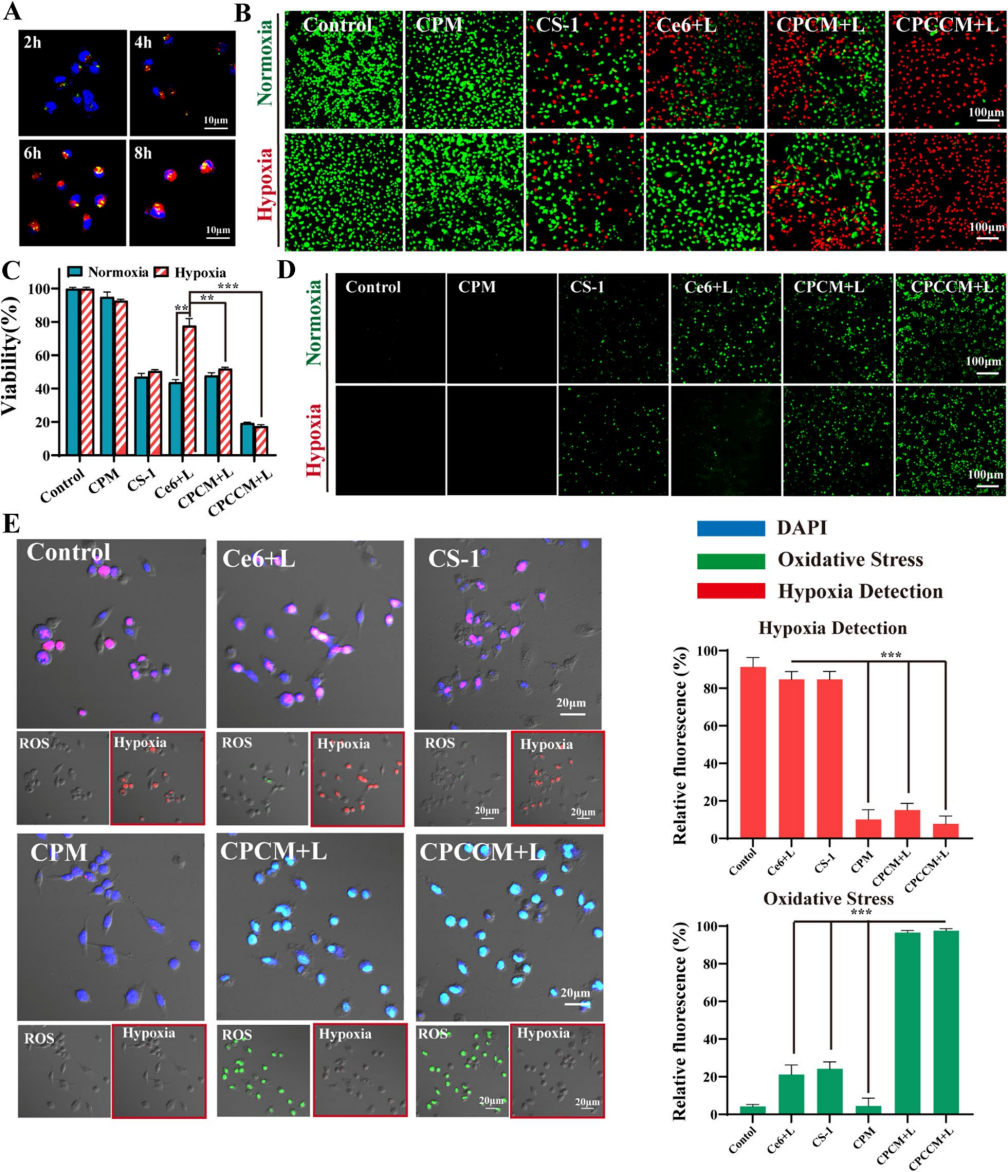
O_2_-enhancing PDT/chemotherapy of CPCCM with laser irradiation (CPCCM + L). **A** CLSM images of MDA-MB-231 cells with CPCCM (50 μg/mL) treatment at 2, 4, 6, 8 h. **B** Live/dead cell staining of CS-1 (5 μg/mL), Ce6 (2.5 μg/mL), CPM, CPCM, and CPCCM (50 μg/mL). **C** Viability of MDA-MB-231 cells treated with CPCCM (50 μg/mL) under 660 nm laser irradiation (200 mW). **D** ROS analysis of CS-1 (5 μg/mL), Ce6 (2.5 μg/mL), CPM, CPCM, and CPCCM (50 μg/mL) under normoxia or hypoxia. **E** The effect of CP@CS-1, CPCM + L, and CPCCM + L (50 μg/mL) treatment on ROS induction. Data were shown as mean ± SD, n = 3. ANOVA was used to assess statistical significance, ***p < 0.001

To evaluate the anticancer activity of CPCCM in vitro, the semi-inhibitory concentration of CS-1 and the optimal irradiation time for CPCCM was firstly investigated. MTT assay demonstrated that the viability of 4T1 and MDA-MB-231 decreased in CS-1 dose-dependent manner with IC_50_ of 4.781 μM and 4.272 μM, respectively (Additional file 1: Fig. S2A, B). In addition, by performing fluorescence imaging of MDA-MB-231 cells incubating with CPCCM NPs at different time points, we found that CPCCM NPs mostly entered the cells after 6 h which indicated the optimal time point of laser irradiation at 6 h (Fig. 6A). Subsequently, CPCCM NPs with optimal CS-1 concentration was used to study its anti-tumor ability by MTT assay, live/dead staining, ROS imaging, and FACS analysis in vitro. Additional file 1: Fig. S3 indicated that the viability of MDA-MB-231 cells with CPCC and CPCC + L treatment were 47.9% and 26.2%. The cell viability after CPCCM + L treatment was slightly increased compared with CPCC + L due to the inhibition of membrane coating on the CS-1 release. After incubation for 48 h, the viability of cells with CPCCM + L treatment was 26.7%. Moreover, the Calcein-AM/Propidium Iodide (PI) staining showed a trend consistent with that of CCK-8 results (Fig. 6B). Compared with free Ce6, the load of CP NPs and the wrapping of the membrane both enhance the PDT efficacy. On the other hand, we studied the changes in ROS levels before and after treatment with different components. Consistent with the results of MTT assay, CPCCM and CPCC NPs detected strong fluorescence signals under laser irradiation. indicating CPCCM and CPCC NPs could efficiency induce ROS production. In addition, CS-1 can induce ROS production, which was consistent with previous reports [25].

To confirm that CPCCM NPs can produce O_2_ under NIR irradiation to improve PDT efficacy by attenuating PDT-induced hypoxia, we performed MTT assay and fluorescence imaging using MDA-MB-231 cells as objects. As shown in Fig. 6C, both free Ce6 and CS-1 showed high toxicity towards MDA-MB-231 cells in normoxia environment. However, their cytotoxicity disappeared under the hypoxia condition without ^1^O_2_ generation. In contrast, similar cytotoxicity of CP (CP@CS-1, CPCM + L, CPCCM + L) was observed in both normoxia and hypoxia. Meanwhile, 2′,7′-dichlorofluorescein diacetate (DCFH-DA), an intracellular ROS sensor, can be hydrolyzed by intracellular esterase and rapidly oxidized by ROS to emit bright green fluorescence. This result revealed that CP NPs can autonomously generate O_2_ to fulfill the requirement of ROS induction in the hypoxic environment. In addition, the ability of CP NPs for attenuating hypoxia improved the efficacy of chemotherapy in vitro.

ROS/ hypoxia detection kit was used to detect intracellular hypoxia and ROS levels. In this kit, the nitro group of the hypoxia detection probe can be reduced to hydroxylamine and amino groups under an intracellular hypoxia, resulting in the appearance of red fluorescence. Meanwhile, DCFH-DA be used to detect ROS to emit bright green fluorescence. Under hypoxic environment, both Ce6 + L and CS-1 treated cells displayed remarkably strong red fluorescence and weak green fluorescence. In contrast, cells with CP NPs treatment only showed weak red fluorescence under hypoxic environment as CP NPs can efficiently attenuate hypoxic condition by generating O_2_. In addition, CPCM + L and CPCCM + L treated cells simultaneously emitted strong green fluorescence (Fig. 6E). This data suggested the enhancement function of O_2_ produced by CP NPs on the efficacy of chemo-PDT therapy.

### In vivo performance of the combined therapy on tumor

**Fig. 7:**
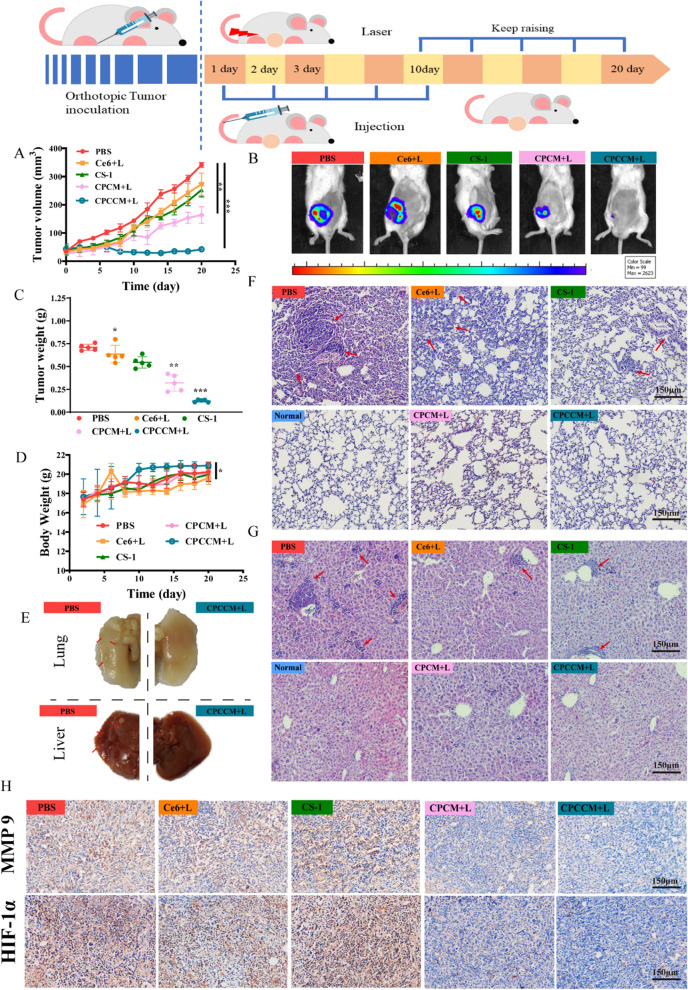
In vivo subcutaneous tumor therapies. **A** The scheme of orthotopic tumor inoculation and dosing regimen (Ce6: 2.5 mg/kg, CS-1: 1 mg/kg, CPCM, CPCCM: 5 mg/kg. laser (L) irradiation: 0.2 W/cm^−2^). **B** Tumor volumes of MDA-MB-231 tumor-bearing mice in different groups. **C** The average tumor weights of mice with different treatments (n = 5). **D** Variation of body weights of tumor-bearing mice with different treatments. **E** Fluorescence photographs of MDA-MB-231-bearing mice with different treatments. **F** Images of orthotopic tumors harvested from the mice from mice with different treatments. **G** H&E and TUNEL staining of tumor sections. Data were shown as mean ± SD, n = 5. ANOVA was used to assess statistical significance. *p < 0.05, **p < 0.01, ***p < 0.001

**Fig. 8:**
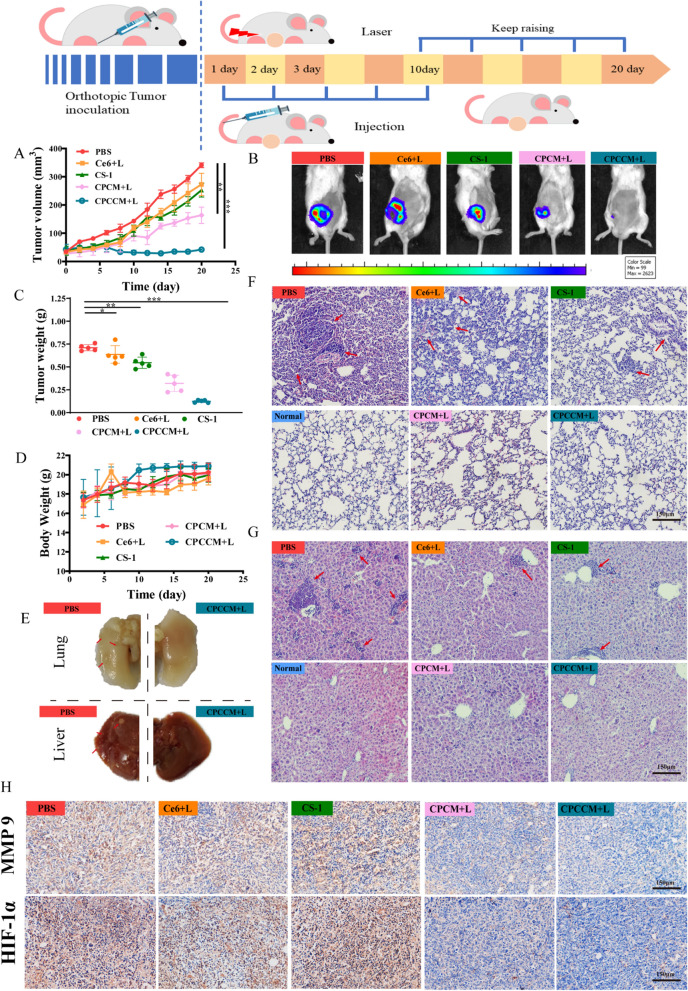
Anti-metastasis ability assay of CPCCM NPs. **A** Tumor growth curves of 4T1 tumor-bearing mice with different treatment. **B** Fluorescence images of 4T1 tumors. **C** The average tumor weight of different groups (n = 5). **D** Variation of body weight of tumor-bearing mice with different treatments. **E** Photographs of liver and lung tissues. **F** H&E staining of the lung with different treatments (the red arrow indicated metastatic nodules). **G** H&E stained the liver with different treatments (the red arrow indicated metastatic nodules). **H** Immuno-histochemistry staining of MMP-9 and HIF-1α in tumor tissues. Data were shown as means ± SD, n = 5. ANOVA was used to assess statistical significance. *p < 0.05, **p < 0.01, ***p < 0.001

As the complexity of the tumor micro-environment will interfere with the theoretical efficacy of NPs, we then tested the performance of the CPCCM under hypoxic tumor microenvironment (Fig. 8A). After 20 days of treatment, the therapeutic effect aiming tumor-bearing mice showed significant differences. The volume growth curves of mice in Fig. 8B showed that all treated components can inhibit tumor growth to a certain extent compared with the control (PBS). However, the CS-1, CC + L, and CPCC NPs treatment groups only partially inhibited tumor growth with relative inhibition rate of 41%, 45.5%, and 52%, respectively. On the contrary, CPCC NPs without laser participation has achieved some therapeutic effects due to its passive targeting ability. After with CPCC + L and CPCCM + L administration, the tumor growth inhibition rate reached about 77.5%. These results demonstrated that the presence of laser and bio-membrane coating makes the combined strategy more efficient. With the assistance of CAT activity of CeO_2_, the tumor inhibition rate under laser irradiation reached 85.5% (Fig. 7C). In addition, the bio-membrane coating further improved inhibitory effects by increasing the drug concentration in the tumor. In addition, we found that CS-1 and Ce6 co-loaded nanomaterials did not cause weight loss of mice. On the contrary, sole CS-1 or Ce6 treatment resulted in weight loss due to systemic toxicity (Fig. 7D). The performing bioluminescence image to monitor tumor volume change of mice with different administration in vivo, we could not find fluorescence signal in some mice treated with CPCCM + L (Fig. 7E), which reflected the significant decrease in tumor volume in the mice. Moreover. the actual images of the tumor further confirmed the reliability of fluorescence image (Fig. 7F).

Furthermore, H&E staining of tumor sections obtained from mice on day 20 showed that CPCC + L and CPCCM + L treatment induced significant tumor necrosis areas and cell nucleus dispersion. In contrast, there were only scattered destroyed areas in other groups (Fig. 7G). In addition, TUNEL staining showed that the number of apoptotic cells in tumor tissue with CPCCM + L treatment is much higher than that of other groups. In our point, the optimal curative effect was attributed to the efficient tumor targeting and penetration ability of CPCCM. In summary, enhanced chemo-PDT efficiency was achieved for MDA-MB-231-bearing mice.

### Inhibition of metastasis induced by CPCCM + L in vivo

Encouraged by anti-tumor in vivo and anti-cell migration in vitro effect, we then asked whether CPCCM can effectively attenuate breast cancer metastasis by inhibiting HIF-1α expression. First, we constructed a metastasis mice model using 4T1 cells to ensure the consistency between tumor origin and the animal. Mice bearing with luciferase-labeled 4T1 tumors in situ were cultured for another 10 days without treatment after administration for 10 days (Fig. 8A). Similar to the above anti-tumor results of Fig. 7, the tumor volume and weight of mice treated with CPCCM + L were well controlled (Fig. 8B–D). Moreover, the tumor volume of 4T1-bearing mice with CPCCM + L administration was ablated to a certain extent on the 6th day, which reflected the excellent performance of the CPCCM + L. Fluorescence images show that the CPCCM NPs demonstrated improve the effects of chem-PDT therapy, and ultimately reduce the viability of 4T1 cells to within the lower limit of fluorescence detection. However, no obvious fluorescence was observed in the lungs and liver due to the small number of viable cells and the short culture time of the animals. By performing morphological observation, we found a lot of tumor metastatic nodules in the liver and lungs (Fig. 8E). the main reason for not observing obvious fluorescence is the absence of tumor metastases. Even so, obvious metastasis inhibition can still be observed in H&E staining sections (Fig. 8F and G). In contrast, PBS treated mice showed large areas of metastatic lesion with condensed nuclei (as shown by the upper left arrow) in the lung. Sporadic metastatic lesions were observed in the CS-1 and CC + L treatment mice. Free drugs and sole PDT can improve tumor metastasis to a certain extent. No obvious tumor metastasis was observed in the three components loading with CeO_2_. Moreover, fewer metastatic lesions near the blood vessels were found in the liver of mice with CPCCM + L treatment, compared with other groups. These results indicated that CPCCM + L effectively controlled the growth of primary tumors and alleviated tumor metastatic burden.

To further explore the anti-metastasis mechanism of CPCCM + L administration, the levels of HIF-1α and MMP-9 were studied due to their tight relation with CS-1, oxygen environment, and tumor metastasis. As shown in Fig. 8H, high levels of HIF-1α in the tumor regions of mice with PBS, CS-1, and CC + L administration were found. In contrast, down-regulation of HIF-1α was observed in the CPCC group, CPCC + L group, and CPCCM + L group. The low expression of HIF-1α demonstrated the relief of sustainable O_2_ supply on the hypoxia environment of tumor. Similarly, MMP-9, another major factor for tumor invasion and metastasis significantly decreased in the CPCCM + L treated mice. These results indicated that the combination of CS-1 with PDT can effectively suppress tumor metastasis by programming tumor hypoxia micro-environment.

### In vivo toxicity evaluation

**Fig.9:**
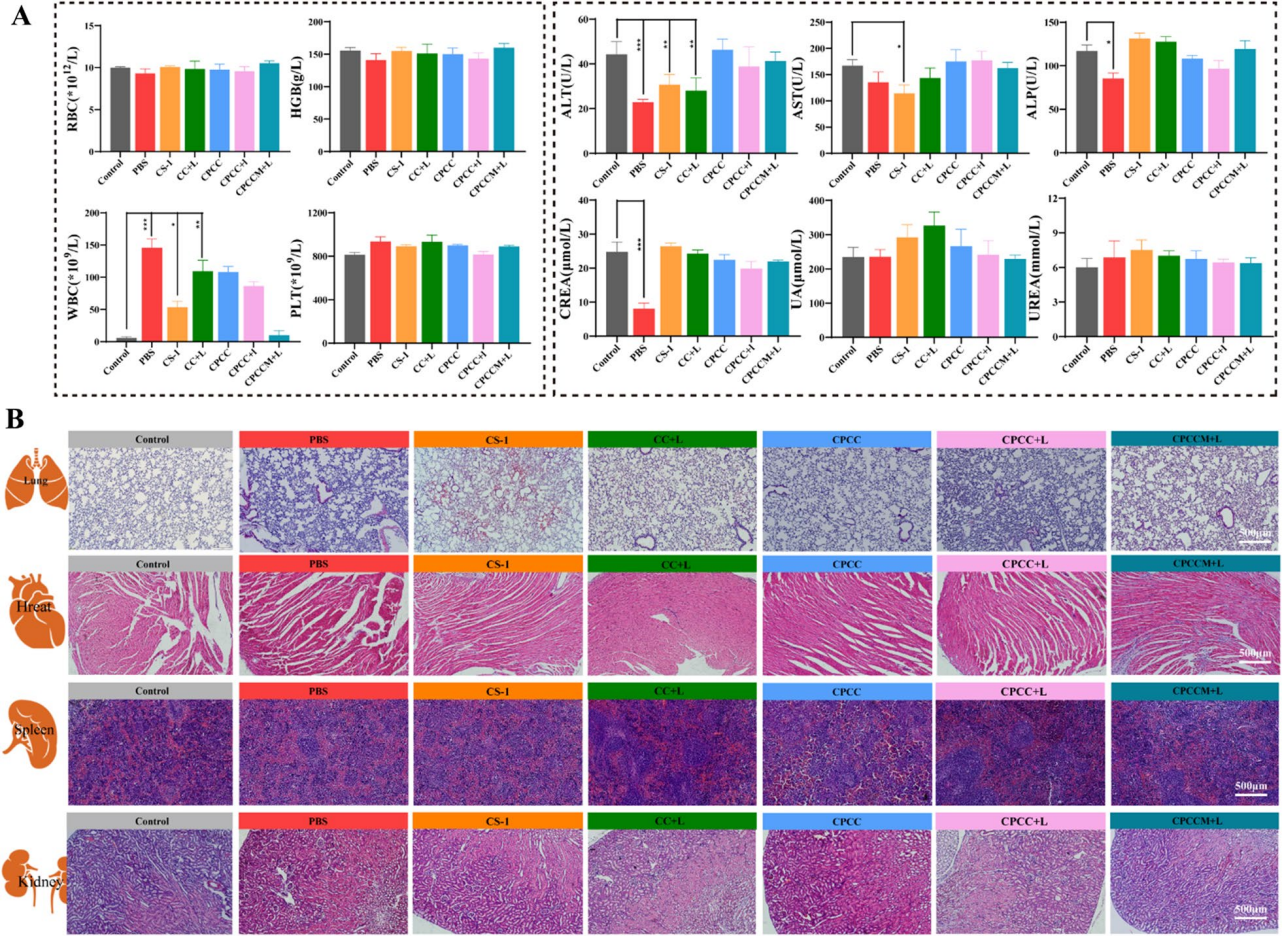
Toxic valuation of mice with different treatments. **A** Whole cell counts and serum biochemical marker analysis of tumor-bearing mice and normal mice. Fresh whole blood and serum were collected at the endpoint of the animal experiment. **B** H&E morphology under different therapies. Major organs were collected at the endpoint of the animal experiment and sectioned for H&E analysis. Data were shown as mean ± SD, n = 3. ANOVA was used to assess statistical significance. *p < 0.05, **p < 0.01, ***p < 0.001

To evaluate the biocompatibility of CPCCM NPs in vivo, the levels of routine blood indicators and biochemical indicators were detected. Compared with normal mice, the indicators of blood cell (RBC), hemoglobin (HGB), and platelets (PLT) of mice with CPCCM + L treatment did not show significant difference, the result of which is similar with in vitro hemolysis and coagulation assay in vitro. However, the levels of white blood cells (WBC) number in the mice increased significantly after different administrations (CPCC, CC + L, CPCC + L). Considering the role of white blood cells in the inflammatory response, this result demonstrated that biomimetic membrane coating can efficiently attenuate the immune reaction. Further attention was paid to the hepatic- and renal-related serum indicators including alanine aminotransferase-ALT, aspartate aminotransferase-AST, and alkaline phosphatase-ALP, uric acid-UA, UREA, creatine kinase-CK, and creatinine-CREA. As we expected, the indicators of liver and kidney function in the mice with CPCCM + L administration are consistent with the normal group. However, the levels of ALT, ALP, and CREA significantly increased in the mice with tumor metastasis. In addition, of both of sole chemotherapy or photodynamic therapy differentially showed the side effects, which was reflected by the increase of ALT and AST levels (Fig. 9A). However, no noticeable signals of major organ damage were observed from the H&E pathological section (Fig. 9B). These results indicated the appreciable in vivo biosafety of CPCCM NPs, which is necessary for future clinical application.

## Conclusions

In summary, a multi-functional nanodrug delivery system based on CPCCM NPs was developed for combinational chemo-PDT therapy against TNBC. The coated hybrid membrane kept the characteristic functions of original cells to endow the homologous targeting ability and long circulation in the blood as well. After accumulating in the tumor regions, CPCCM NPs can effectively consume the excess H_2_O_2_ to generate O_2_ through SOD/CAT-like catalysis. Meanwhile, under NIR irradiation, it acts as a substrate for photosensitizers, resulting in enough highly toxic ^1^O_2_ to attack tumors. In addition, CS-1 showed strong inhibitory effect on the in situ growth of TNBC and distant metastasis to the lung and liver. In vivo study proved the strong anti-tumor function of CPCCM NPs with an inhibitory rate of 95%. In summary, the therapeutic strategy of CPCCM NPs using CeO_2_ nanozyme activity and photosensitizer to enhance ROS generation can not only take advantages of complex tumor micro-environment but also can effectively combat cancer cells during metastasis.

## Supplementary Information

**Additional file 1: Figure S1.** A. CS-1 loading capacity and encapsulation efficiency of CPCCM NPs. B. Ce6 loading capacity and encapsulation efficiency of CPCCM NPs. **Figure S2.** Cell viability assay. A. Viability of 4T1 cells treated with CS-1. B. Viability of MDA-MB-231 cells treated with CS-1. The concentrations of CS-1 are 0, 1, 2.5, 5, 10, 15, and 20 μM, respectively. **Figure S3.** Viability of MDA-MB-231 cells treated with CS-1, Ce6, CPCC, and CPCCM with/without laser after 24/48 h. L represents 660 nm laser irradiation.
