# Supplementary material for: Bio-nanocomplexes with autonomous O2 generation efficiently inhibit triple negative breast cancer through enhanced chemo-PDT
Source: J Nanobiotechnology. 2022 Nov 24;20:500. doi: 10.1186/s12951-022-01706-0 (PMC9694858; doi:10.1186/s12951-022-01706-0)
Supplement: Supplementary file 1 — Additional file 1: Figure S1. A. CS-1 loading capacity and encapsulation efficiency of CPCCM NPs. B. Ce6 loading capacity and encapsulation efficiency of CPCCM NPs. Figure S2. Cell viability assay. A. Viability of 4T1 cells treated with CS-1. B. Viability of MDA-MB-231 cells treated with CS-1. The concentrations of CS-1 are 0, 1, 2.5, 5, 10, 15, and 20 μM, respectively. Figure S3. Viability of MDA-MB-231 cells treated with CS-1, Ce6, CPCC, and CPCCM with/without laser after 24/48 h. L represents 660 nm laser irradiation. [file 12951_2022_1706_MOESM1_ESM.docx]

Additional file 1:
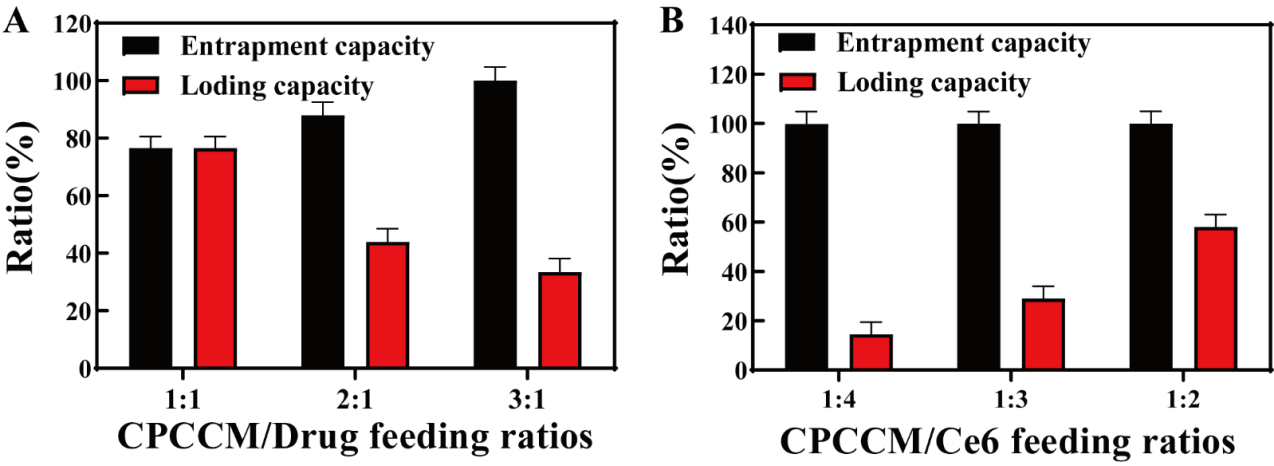


**Fig.S1.** A. CS-1 loading capacity and encapsulation efficiency of CPCCM NPs. B. Ce6 loading capacity and encapsulation efficiency of CPCCM NPs.

**
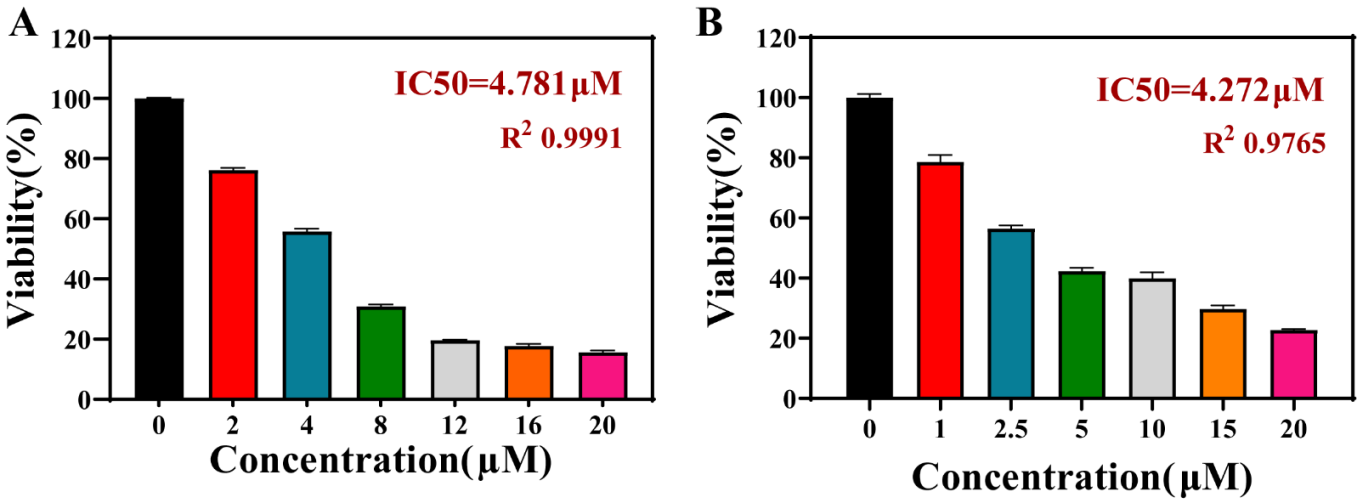
**

**Fig. S2. Cell viability assay** A. Viability of 4T1 cells treated with CS-1. B. Viability of MDA-MB-231 cells treated with CS-1. The concentrations of CS-1 are 0, 1, 2.5, 5, 10, 15, and 20 μM, respectively.

**
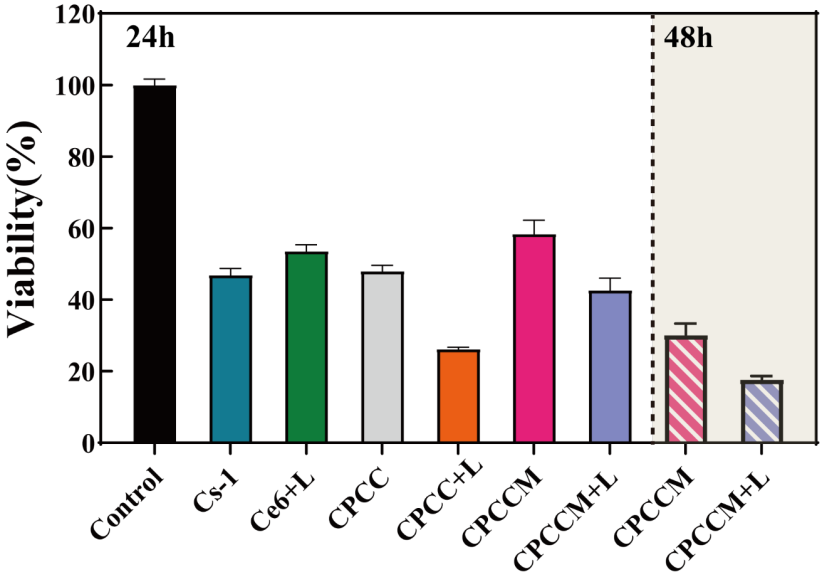
**

**Fig. S3.** Viability of MDA-MB-231 cells treated with CS-1, Ce6, CPCC, and CPCCM with/without laser after 24/48h. L represents 660 nm laser irradiation
